# Annual Meeting of the Medical Society of the County of Erie

**Published:** 1859-02

**Authors:** 


*The Annual Meeting of the Medical Society of the County of Erie* was held in this city, on Tuesday, January the 11th, at the rooms of the Buffalo Medical Association.

In the absence of the President, Prof. Flint, the Vice President, Dr. L. P. Dayton presided, calling the meeting to order at 11 o'clock A. M.

The attendance was very large, a goodly number from the country being present. The transactions of the day were, however, marked with nothing of any very especial public interest. The ordinary business of the Society, only, was gone through with.

Drs. Rathbone, Whitaker, and Mead, were elected members of the Society upon their application, upon the conditions of their complying with the laws of the State, and with the by-laws of the society, in all things therein laid down, in reference to membership.

Dr. C. C. F. Gay delivered the Oration. Subject: ERYSIPELAS.

By vote, the society ordered its publication in the pages of this Journal.

Dr. John P. Cole was appointed *Orator* for the June meeting, and Dr. George Abbott his substitute.

The society elected the following gentlemen to fill the respective offices for the ensuing year:

| | |
|---|---|
| Dr. L. P. Dayton, . . . . | President. |
| " James M. Newman, . . . . . | Vice President. |
| " Jame S. Hawley, . . . . . | Secretary. |
| " C. C. F. Gay, . . . . . . | Treasurer. |
| " James B. Samo, . . . . . | Librarian. |

*Primary Board.*—Drs. S. Eastman, J. Hauenstein, J. F. Miner.

| | | | |
|---|---|---|---|
| *Censors.*—Dr. B. H. Lemon, | Exam. | in | Anatomy and Physiology. |
| " Wm. Gould, | " | " | Pract. Med. and Obstetrics. |
| " C. B. Hutchins, | " | " | Chemistry and Pharmacy. |
| " Wm. Ring, | " | " | Mat. Med. and Botany. |
| " L. J. Ham, | " | " | Med. Jurisp. and Pathology. |

The society had its annual dinner at the American Hotel, sitting down at about 4½ o'clock, to the number of some forty members, and spent several of the succeeding hours in a most pleasant manner, the interchange of kindly sentiments upon the occasion, serving, most certainly, in no manner to weaken any of the ties of friendship which existed among those who partook of these festivities; and, if any regrets accompanied or followed the feast of good things at the board, it was for those, who, from any cause, were not partakers with their fellows in the kindly intercourse of the occasion. May all who were present upon this occasion be permitted a reunion at the next annual meeting, and with them, the absent ones from this festival. *

